# Alterations in ZnT1 expression and function lead to impaired intracellular zinc homeostasis in cancer

**DOI:** 10.1038/s41420-019-0224-0

**Published:** 2019-11-12

**Authors:** Adrian Israel Lehvy, Guy Horev, Yarden Golan, Fabian Glaser, Yael Shammai, Yehuda Gérard Assaraf

**Affiliations:** 1 The Fred Wyszkowski Cancer Research, Laboratory, Department of Biology, Technion-Israel Institute of Technology, Haifa, Israel; 2 Bioinformatics Knowledge Unit, The Lorry, I. Lokey Interdisciplinary Center for Life, Sciences and Engineering, Technion-Israel, Institute of Technology, Haifa, Israel

**Keywords:** Cancer, Cancer, Molecular biology

## Abstract

Zinc is vital for the structure and function of ~3000 human proteins and hence plays key physiological roles. Consequently, impaired zinc homeostasis is associated with various human diseases including cancer. Intracellular zinc levels are tightly regulated by two families of zinc transporters: ZIPs and ZnTs; ZIPs import zinc into the cytosol from the extracellular milieu, or from the lumen of organelles into the cytoplasm. In contrast, the vast majority of ZnTs compartmentalize zinc within organelles, whereas the ubiquitously expressed ZnT1 is the sole zinc exporter. Herein, we explored the hypothesis that qualitative and quantitative alterations in ZnT1 activity impair cellular zinc homeostasis in cancer. Towards this end, we first used bioinformatics to analyze inactivating mutations in ZIPs and ZNTs, catalogued in the COSMIC and gnomAD databases, representing tumor specimens and healthy population controls, respectively. ZnT1, ZnT10, ZIP8, and ZIP10 showed extremely high rates of loss of function mutations in cancer as compared to healthy controls. Analysis of the putative functional impact of missense mutations in ZnT1-ZnT10 and ZIP1-ZIP14, using homologous protein alignment and structural predictions, revealed that ZnT1 displays a markedly increased frequency of predicted functionally deleterious mutations in malignant tumors, as compared to a healthy population. Furthermore, examination of ZnT1 expression in 30 cancer types in the TCGA database revealed five tumor types with significant ZnT1 overexpression, which predicted dismal prognosis for cancer patient survival. Novel functional zinc transport assays, which allowed for the indirect measurement of cytosolic zinc levels, established that wild type ZnT1 overexpression results in low intracellular zinc levels. In contrast, overexpression of predicted deleterious ZnT1 missense mutations did not reduce intracellular zinc levels, validating eight missense mutations as loss of function (LoF) mutations. Thus, alterations in ZnT1 expression and LoF mutations in ZnT1 provide a molecular mechanism for impaired zinc homeostasis in cancer formation and/or progression.

## Introduction

Zinc is vital for the structure and function of ~10% of the human proteome. Intracellular zinc levels are tightly regulated by zinc transporters, termed ZIPs and ZnTs^1^. Zinc is essential for the structure and function of multiple proteins involved in DNA replication, gene expression, redox signaling, cell cycle regulation, and apoptosis^2,3^. Since most intracellular zinc is either compartmentalized or associated with proteins, free intracellular zinc levels are in the picomolar to low nanomolar range^4^, and thus tight regulation is necessary to maintain zinc homeostasis^1,4^. ZIPs import zinc into the cytosol from the extracellular milieu through the plasma membrane, or translocate it from the lumen of organelles into the cytoplasm, whereas ZnTs sequester zinc within organelles or export it to the extracellular milieu^4^. Furthermore, non-specific metal-binding proteins termed metallothioneins (MTs) bind and buffer zinc levels in the cytoplasm^5^.

Notably, ZnT1 is the sole zinc efflux transporter residing in the plasma membrane, and is functionally non-redundant in the maintenance of zinc homeostasis^4^. Unsurprisingly, homozygous ZnT1 gene knockout is embryonic lethal in mice, while embryos of heterozygous female mice developed abnormally under zinc deficient conditions^6^. Moreover, increased ZnT1 protein levels have been observed in mammalian cells that have acquired a zinc resistance phenotype^7^. The aforementioned research shows that ZnT1 knockout or overexpression functionally alter zinc homeostasis and development.

Impaired zinc homeostasis has been observed in various cancers^8^. Metadata analyses revealed significantly decreased serum zinc levels in breast, prostate, liver, lung and kidney cancer^8^. Alterations in the transcript levels of zinc transporters were previously reported in relation to cancer. For example, overexpression of ZIP6 and ZIP10 has been implicated in breast cancer progression^9^. Moreover, downregulation of ZnT5 and ZnT6 mRNA is observed in the early stages of prostate cancer, and ZnT9 mRNA upregulation was observed in stage II of prostate cancer tumors^10^. Significant upregulation of ZnT1 mRNA was also observed in prostate cancer tissue, independent of tumor stage^10^. Recently, overexpression of ZnT1 mRNA has been associated with the development and progression of bladder cancer^11^. While dysregulation of zinc transporter expression is observed in cancer, there is no clear evidence that zinc transporter mutations contribute to cancer progression per se.

The availability of high throughput data regarding mutagenesis and expression patterns in tumor specimens, and the advancement in mutational annotation methods allows for the identification of potentially novel cancer driver genes^12^. In the present study, we identified ZnT1 as a potential novel cancer driver gene. To this end we first utilized the COSMIC database^13^ of somatic mutations in cancer, and the gnomAD database^14^, as a comparative control, since it provides healthy exome and genome sequences. To analyze ZnT1 expression data, we used the Gene Expression Profiling Interactive Analysis (GEPIA)^15^ website, based on TCGA and GTEx data. We found increased ZnT1 transcript levels in clinical tumor specimens, as well as an increase in the relative frequency of ZnT1 mutations in tumor specimens, compared to other ZnTs and ZIPs. We show the consequent impairment of zinc homeostasis via functional validation of ZnT1 overexpression, as well as of inactivating ZnT1 mutations that occur in cancer, thus providing a mechanistic role for ZnT1 alterations in the impairment of zinc homeostasis in cancer.

## Materials and methods

### Classification of mutations

Mutations identified in ZnT1-10 and ZIP1-14, in cancer specimens from the COSMIC database, and healthy control populations from the gnomAD database, were analyzed using the variant effect predictor (VEP)^16^ for mutation effect analysis. Effect of missense mutation was predicted with PolyPhen-2^17^, which is part of the VEP. The odds of predicted “benign”, “possibly deleterious”, and “probably deleterious” mutations were compared for each zinc transporter gene in the healthy population and cancer specimens. Hypothesis testing was performed with the Fischer Exact test. Correction for multiple hypotheses was performed using FDR (False Discovery Rate) with *α* = 0.05. ConSurf^18^ was used for an amino acid sequence-based analysis of residue conservation; the ConSurf server assigns a conservation score of 1-9, based on an algorithm which relies on homologous sequence conservation. Residue conservation among ZnTs was determined by multiple sequence alignment of ZnT1-ZnT10.

### ZnT1 expression analysis

We used GEPIA^15^ to explore ZnT1 gene expression, and Kaplan–Meier survival analysis in GTEx (REF) and TCGA in 30 cancer types. For gene expression analysis, we used a p-value cutoff of 0.005. The cox proportional hazard ratio and the 95% confidence interval information are included in the survival plot^15^.

### Predicted topology and structure

The predicted topology of ZnT1 was generated via Protter^19^, with the transmembrane regions pre-defined via TOPCONS^20^, a web server for the consensus prediction of membrane protein topology by combining several transmembrane prediction tools.

### Modeling 3D structure

Given the very low sequence identify between ZnT1 and its closest PDB homologue, YiiP (3j1z), we executed the modeling in a two stage approach, which combines the RaptorX^21^ method for the template identification and MEDELLER^22^, a homology-based coordinate generation method optimized to build highly reliable TM protein models.

The RaptorX method is a tool for the detection of remotely related template sequences which produces high-quality template-target alignment by means of a nonlinear context-specific alignments potential and probabilistic consistency algorithm^21^. We used RaptorX to obtain a pairwise alignment between ZnT1 and YiiP template, a cation diffusion transporter in *Shewanella oneidensis*, for which a PDB structure has been solved (3j1z). The initial alignment produced by RaptorX was manually improved (Fig. S1) with the help of Jalview^23^, and then input it into MEDELLER, a Homology-Based Coordinate Generation for Membrane Proteins software. The high quality of MEDELLER’s core models is achieved by reliably selecting parts of the template structure^24^. MEDELLER generates two 3D models, a “high accuracy” model which predicts the 3D positions and orientation of TM helices, and a “high coverage” model, which adds the loops connecting the helices.

### Cell culture and transient transfections

HEK-293 cells grown in RPMI-1640 medium containing 10% fetal calf serum (FCS), were used for all functional validation experiments (Rhenium, Modi’in Makabim-Re’ut, Israel). Transient transfections were performed at 60% cell confluence, 48 h post-seeding, using the Jet-PEI transfection protocol, as previously described^25^. For confocal microcopy experiments, we used 24-well glass-bottom plates, where cells were transfected with 0.25 µg Ruby-tagged ZnT1 plasmids, supplemented with ZnT2-HA plasmids to a total DNA amount of 1 µg. For flow cytometry experiments, cells were seeded in 24-well plates and transfected with 0.25 µg Ruby-tagged ZnT1 plasmid, supplemented with ZnT2-HA plasmid to a total amount of 1 µg DNA. For qRT-PCR experiments, cells were seeded in 6-well plates, and transfected with 0.75 µg ZnT1-Ruby constructs, supplemented to 3 µg with pcDNA3.1+ empty vector plasmid. The control transfection contained 3 µg pcDNA3.1+ empty vector.

### Construction of expression vectors

A pcDNA3.1 Zeo (+) expression plasmid encoding for the WT ZnT1 tagged with a red fluorescent Ruby protein was generated via restriction enzyme digestion of a YFP-tagged ZnT1 pcDNA3.1 Zeo (+) vector by *KpnI* and *XhoI*, and ligation of the ZnT1 insert into a Ruby-tagged pcDNA3.1 Zeo(+) vector, obtained by restriction enzyme excision of the Ruby tag from ZnT2-Ruby by *KpnI* and *XhoI*, using previously described methodology^26^. The mutations were introduced into the ZnT1‐Ruby expression vector using *Pfu Turbo* DNA polymerase (QuikChange kit; Stratagene, La Jolla, CA, USA) and the oligonucleotide primers are listed in Table S1.

### Flow cytometry

Twenty-four hours after transfection, HEK-293 cells were treated with 10 µM zinc sulfate, and 0.5 µM FluoZin3 in RPMI-1640 containing 10% FCS for 1 h at 37 °C. Cells were then washed with PBS, trypsinized and re-suspended in PBS at a density of 6.66 × 10^5^ cells/mL. Ten thousand events were analyzed by flow cytometry via the FACS Aria IIIU cell sorter (BD Biosciences 2350 Qume Drive San Jose, CA 95131) for FluoZin3 fluorescence within the population of Ruby-positive cells, using FCS express software (De Novo Software, Glendale, CA, USA). A two-tailed, paired *t*-test was performed, comparing the raw FluoZin3 fluorescence values to ZnT2-HA and WT ZnT1 Ruby co-transfectant (Table S4).

### Fluorescence microscopy

Twenty-four hours after transfection, HEK-293 cells were treated with 30 µM zinc sulfate, and 1 µM FluoZin3 in growth medium for 1 h at 37 °C. Cells were washed with growth medium, and Hoechst 33342 (2 µg/mL) staining was applied for 10 min. Cells were washed with growth medium and left in the same growth medium for the microscopy imaging. Live cells were imaged using a Zeiss Confocal 710 inverted microscope (Thornwood, NY, USA) at x63 magnification under immersion oil. Laser excitation parameters were 488 nm for FluoZin3, and 543 nm for Ruby; respective lasers were kept at constant parameters throughout all experiments

### q-RT-PCR

Twenty-four hours post-transfection, cells were treated with 50, 60, or 75 µM zinc sulfate in growth medium and incubated for 2 h at 37 ^o^C. RNA extraction and cDNA synthesis was performed as previously described^27^. qRT-PCR was performed, using primers targeted for MT-2, as seen below.

MT-2 forward: 5′ GCA CCT CCT GCA AGA AAA GCT 3′

MT-2 Reverse: 5′ TTT GTG GAA GTC GCG TTC TTT A 3′

## Results

### Increased frequency of predicted deleterious ZnT1 mutations in cancer samples, compared to a healthy population

We first assessed whether alterations in zinc transporter function are associated with cancer. Toward this end, we compared the rates of both loss of function (LoF) and missense mutations, between COSMIC and gnomAD representing cancer specimens and healthy population controls, respectively, in both ZnT1-10 as well as ZIP1-14. LoF mutations were defined as stop/start codon gained/lost, frameshift, and splice variants. Missense mutations underwent classification with PolyPhen-2 to predict their potential to be functionally deleterious (Fig. S2). The resulting predictions were grouped into two categories, “probably damaging”-predicted mutations which were defined as “deleterious”, whereas “possibly damaging” and “benign”-predicted mutations which were defined as “non-deleterious”.

**Fig. 1:**
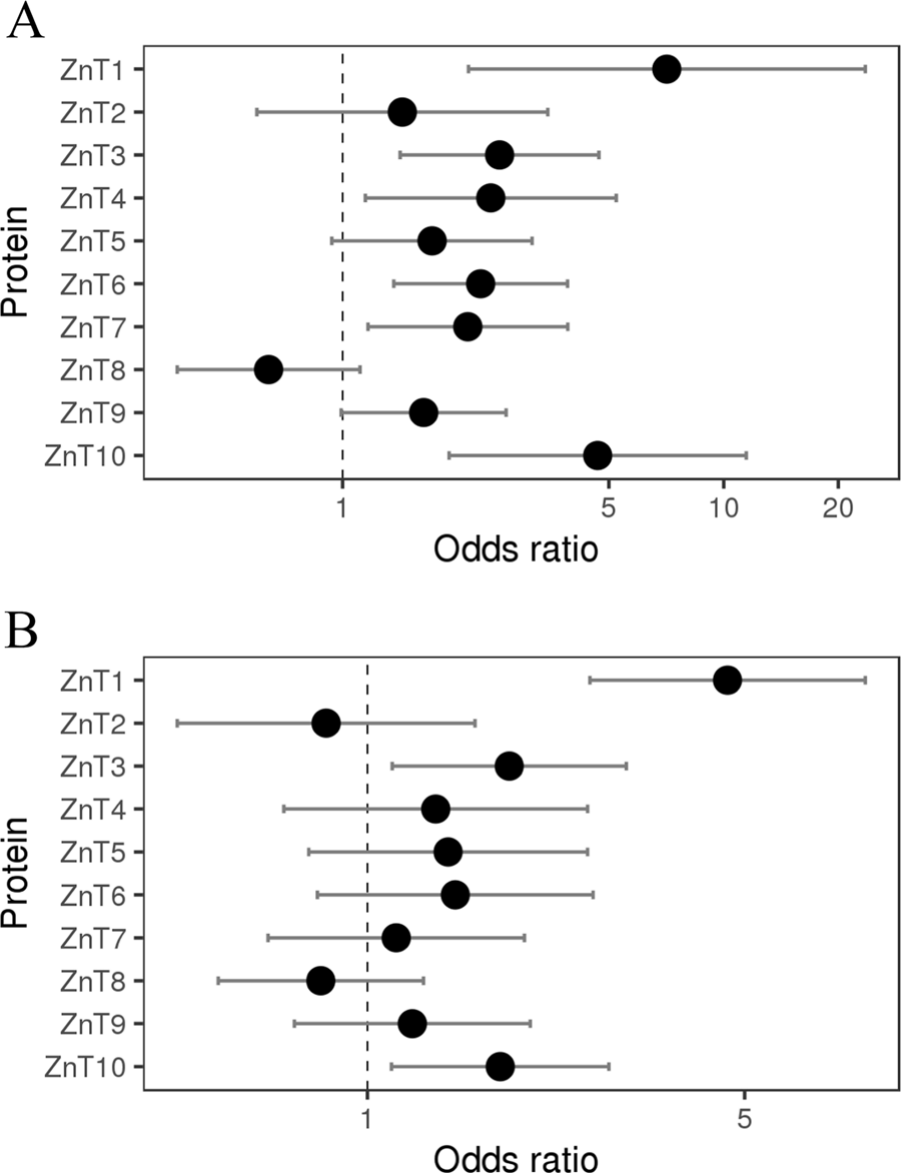
Inactivating mutations in ZnT zinc transporters are more abundant in cancer as compared to healthy controls. Odds ratio (black dot) of **a** LoF mutations and **b** predicted deleterious missense mutations, identified in ZnT1-ZnT10 in tumor samples (COSMIC) versus healthy controls (gnomAD). Error bars represent 0.95 confidence interval

The relative frequencies of LoF mutations in ZnT1-10 (Fig. 1a, Table S2) and ZIP1-14 were calculated for cancer specimens and healthy controls (Fig. S3, Table S3). Among ZnTs, ZnT1 and ZnT10, and to a lesser degree, ZnT3, ZnT6 and ZnT7, had a significant increase in LoF mutations in cancer samples, when compared to healthy control tissues (Fig. 1a, Table S2). Among ZIPs, ZIP10 had the most significant increase in LoF mutations, and to a lesser extent, ZIP8 and ZIP14 (Fig. S3, Table S3). Regarding missense mutations of ZIPs in tumor specimens, only ZIP5, had a significant increase (Fig. S3b, Table S3). Notably, among both ZIPs and ZnTs, ZnT1 had the most significant increase in the frequency of predicted deleterious missense mutations, being highly statistically significant with a p-value of 2.46 × 10^−7^ (Fig. 1b and Table S2). The significantly higher ratio of LoF and predicted deleterious ZnT1 mutations in cancer specimens suggests that LoF ZnT1 mutations, and consequently impaired zinc homeostasis, may possibly contribute to oncogenesis and/or cancer progression.

### Overexpression of ZnT1 predicts decreased survival of cancer patients

**Fig. 2:**
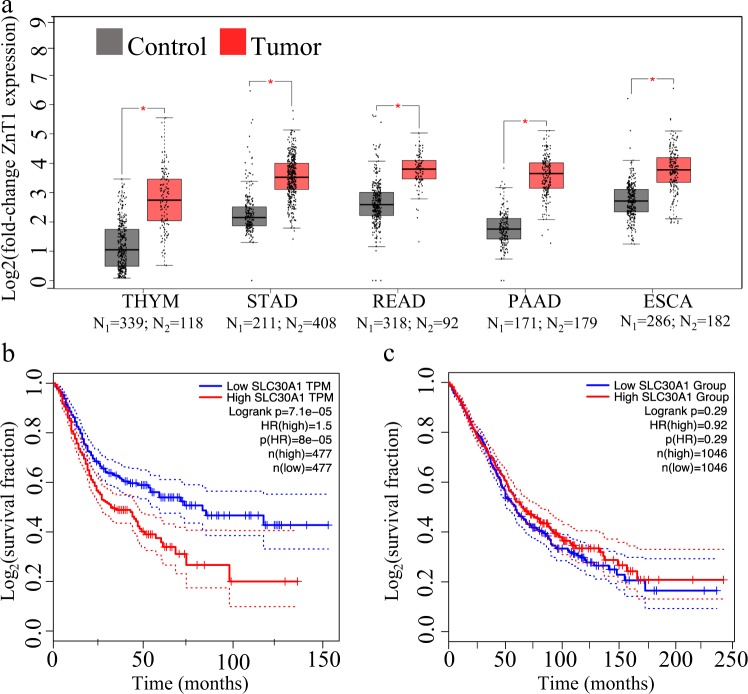
ZnT1 overexpression predicts dismal prognosis in cancer. GEPIA expression and survival analyses are presented. **a** Boxplot representation of ZnT1 expression in five distinct cancer types and healthy controls. Asterisk indicates *p*-value < 0.005 (ANOVA). THYM-thymoma, STAD-stomach adenocarcinoma, READ-rectal adenocarcinoma, PAAD-pancreatic adenocarcinoma, and ESCA-esophageal carcinoma. **b** Kaplan–Meier survival plot in the cancers presented (**a**). **c** Kaplan–Meier survival plot in five types of cancer (thyroid cancer, ovarian cancer, brain glioma, glioblastoma, and colon adenocarcinoma) with no significant tendency to ZnT1 overexpression. HR signifies hazard ratio. Dashed lines in (**b**), (**c**) represent the 95% confidence interval

We further explored ZnT1 transcript levels in cancer specimens. We used GEPIA to compare ZnT1 levels in 30 cancer types and their cognate healthy tissues (all cancers are available in GEPIA excluding Pheochromocytoma and Paraganglioma that did not have sufficient control samples for ZnT1). We found significantly increased ZnT1 mRNA levels (*p* < 0.005, ANOVA) in esophageal carcinoma (ESCA), pancreatic adenocarcinoma (PAAD), rectum adenocarcinoma (READ), stomach adenocarcinoma (STAD), and thymoma (THYM) (Fig. 2a), as well as significantly decreased ZnT1 expression levels in Kidney Chromophobe-type (KICH) renal cell cancer (Fig. S4). We further analyzed cancer patient survival rates in the group of cancers with significant ZnT1 overexpression. Remarkably, patient survival decreased significantly when compared to the low ZnT1 level control group (Fig. 2b). For comparison, in thyroid cancer, ovarian cancer, brain glioma, glioblastoma, and colon adenocarcinoma, where ZnT1 transcript levels were not significantly different, patient survival rates did not differ (Fig. 2c). Thus, increased ZnT1 transcript levels appears to be an independent parameter for dismal prognosis in several cancers.

### The structural implications of ZnT1 missense mutations selected for functional validation

**Fig. 3:**
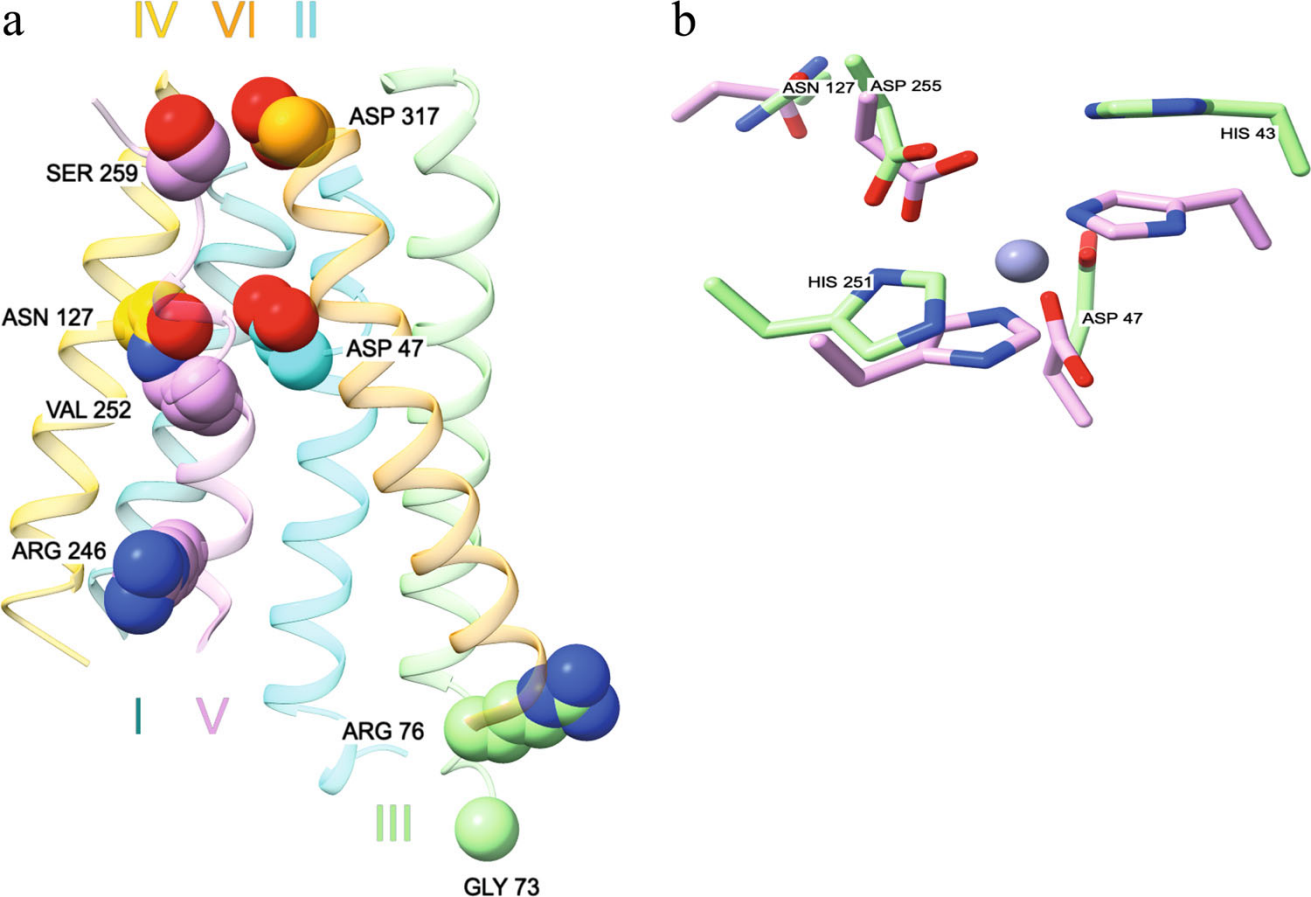
The structure model of ZnT1 and the implications of inactivating missense mutations. **a** Structural prediction of the transmembrane regions of ZnT1, using RaptorX alignment, and then the MEMOIR modeling server, with the bacterial *Shewanella oneidensis* zinc transporter 3j1z as a template. The residues selected for functional validation are represented as spheres. The roman numerals signify the number of the TM helix. **b** Structural superposition of zinc-binding site A in ZnT1 (green residues) and ZnT2 (violet residues), with a zinc ion (purple) from the YiiP structure

We selected eight ZnT1 LoF missense mutations for functional validation (Table 1), based on predicted protein structure of ZnT1 (Fig. 3), PolyPhen-2 scores, Consurf conservation scores, 2D topology (Fig. S5), and published data. The eight residues selected for functional validation reside along the transmembrane (TM) helices of the predicted ZnT1 structure, or at the beginning of their loops (Fig. 3). ZnT1 contains the canonical HD-DH zinc-binding motif, conserved among zinc transporters^28^. Notably, when comparing the 3D positions of the zinc-binding motif (Site A)^29^, in the ZnT1 and ZnT2 models, as well as with the YiiP equivalent Fe/Zn binding site (PDB 3H90), we found similar 3D organization (Fig. 3b). Given that we have previously shown that the ZnT2 model, which is also based on YiiP, is able to predict the zinc permeation pathway along the transporter^29^, and given the very low homology between ZnT1 and ZnT2 (less than 20% amino acid identity), we believe the similarity in Site A is a good indication of the new ZnT1 model validity, and, in consequence, can be used to rationalize the impact of each of the eight mutants found by this study on ZnT1. Thus, we present here the resulting high-accuracy partial 3D model of ZnT1, focusing on the TM helices, and, using the model, we attempt to rationalize the structural and functional impact of the eight mutations tested in this study, as follows (Fig. 3).

**Table 1:** Mutations in ZnT1 selected for functional validation, and their respective predicted structural properties, PolyPhen-2, Consurf, and literature comparisons

Mutation	Topology	PolyPhen-2 score	Consurf score
D47G	TM helix II	1	8
G73D	Intracellular loop 1	1	9
R76L	TM helix III	0.997	9
N127K	TM helix IV	0.849	9
R246C	TM helix V	0.999	9
V252G	TM helix V	0.999	9
S259L	Extracellular loop 3	0.998	9
D317Y	Extracellular loop 3	1	9

D47 is part of the consensus zinc-binding motif^28^ in ZnT1 TM helix II, in consequence, its mutation to glycine will disrupt zinc transport. The N127 residue (equivalent to N189 in ZnT2) in TM helix IV is less than 10 Å away from approximate location of the zinc ion in the zinc permeation pathway (obtained by superimposition ZnT1 model with 3H90), and its mutation to positively charged lysine strongly suggests a functional impairment for zinc transport (Fig. 3b). Arginine mutants to hydrophobic R76L and uncharged R246C residues, on TM helices III and V, respectively, have most probably a destabilizing effect on the membrane anchoring of ZnT1^30^. The G73D mutation occurs in a short loop between TM helix III and TM helix IV and may reduce loop flexibility, and therefore destabilize the overall structure^31^. In addition, the V252G mutation in TM helix V may disrupt the core helical organization by decreasing the hydrophobicity in the inter-helical region, or causing helix disruption on the core of TM helix organization, most probably resulting in a structural destabilization of the helix core^32^. Mutations S259L and D317Y replace polar and charged residues serine and aspartate with highly hydrophobic leucine and tyrosine. Although these mutations are located in less accurately modeled regions of our model (i.e., loops), their substitution by hydrophobic moieties and spatial proximity suggests that they can have a similar disruptive impact on helix organization, membrane-protein anchoring or both^30,31^.

### Wild type (WT) ZnT1 overexpression but not deleterious mutant ZnT1 overexpression results in low intracellular zinc levels

**Fig. 4:**
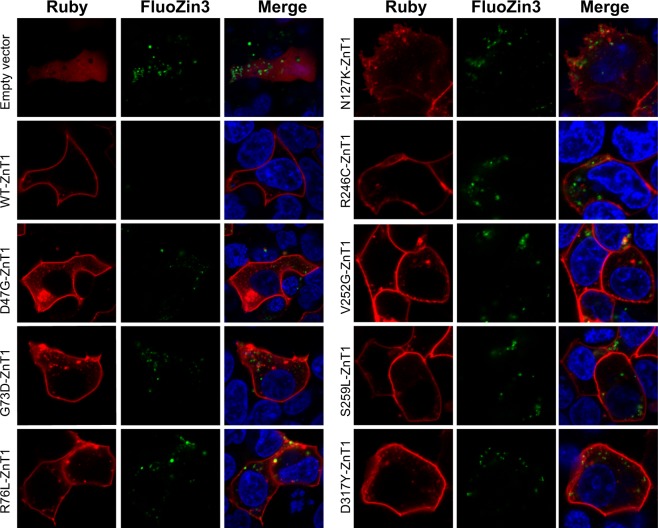
Low vesicular zinc accumulation in cells overexpressing WT ZnT1 but not in cells overexpressing predicted deleterious ZnT1 mutants. HEK-293 cells co-transfected with either WT ZnT1 and ZnT2-HA, or with predicted deleterious ZnT1 mutations and ZnT2-HA. Cells transfected with WT ZnT1 display extremely low FluoZin‐3 fluorescence (green fluorescence) that indicates vesicular zinc accumulation, while cells co-transfected with predicted deleterious ZnT1 mutants display relatively high vesicular FluoZin3 levels that are similar to empty vector. Red fluorescence represents the WT- or mutant ZnT1‐Ruby, or the empty-vector-Ruby (panel 1), validating that transfection was efficient in all mutants. Hoechst 33342 (blue fluorescence) was used to label nuclear DNA. A magnification of ×63 under immersion oil was used. The 488 nm laser remained constant in all the photographs in order to be able to properly compare cellular FluoZin‐3 levels

We explored the functional consequences of WT ZnT1 overexpression and predicted deleterious ZnT1 mutations on intracellular zinc. To test the impact of transient overexpression of WT or predicted deleterious ZnT1 missense mutations on intracellular zinc levels, we developed a novel functional assay where ZnT2, which sequesters zinc within intracellular vesicles, is transiently co-overexpressed along with either empty vector, as a negative control, or WT or mutant ZnT1. Intracellular zinc levels were then assessed by quantification of the fluorescence levels of the zinc probe, FluoZin3. Therefore, in all the experiments that assess intracellular zinc levels, the non-fluorescently tagged ZnT2-HA protein was co-expressed in the cells in order to measure vesicular zinc accumulation. Confocal fluorescence microscopy imaging revealed that cells transiently overexpressing WT ZnT1 exhibit very low cellular FluoZin3 fluorescence levels, when compared to the control cells transfected with empty vector, which expressed only the endogenous WT ZnT1. This result indicates that ZnT1 overexpression leads to low intracellular zinc accumulation (Fig. 4). Conversely, cells transiently overexpressing predicted deleterious ZnT1 mutants exhibit an increase in intracellular zinc levels, when compared to cells which overexpressed WT-ZnT1 (Fig. 4). Overexpression of LoF ZnT1 mutants showed similar levels of intracellular zinc when compared to the empty vector control, indicating that these mutants fail to export intracellular zinc. These results confirm that the eight ZnT1 missense mutations studied here are functionally deleterious. Importantly, the ZnT1 mutants preserved their canonical plasma membrane localization (Fig. 4) and ZnT2 co-transfection did not affect ZnT1 localization to the plasma membrane, as we have previously shown^33^.

**Fig. 5:**
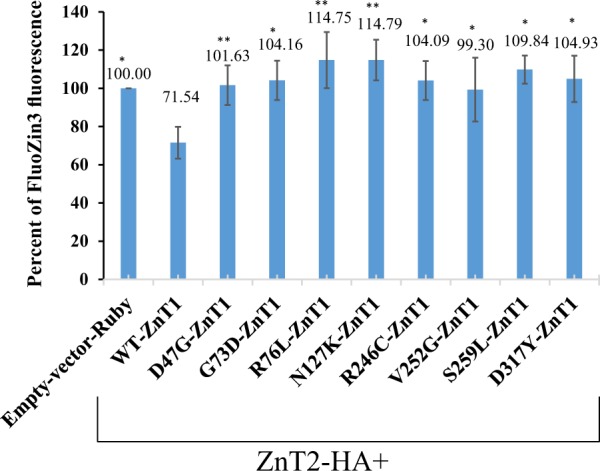
Low vesicular zinc accumulation in cells overexpressing WT ZnT1 but not in cells overexpressing predicted deleterious ZnT1 mutants. Quantitative analysis of Fig. 4. Percent Fluozin3 fluorescence is relative to ZnT2-HA and empty vector-Ruby. HEK-293 cells co-transfected with mutant ZnT1 and WT ZnT2, and treated with 30 µM zinc sulfate, display increased vesicular FluoZin3 levels compared to cells co-transfected with WT ZnT1 along with ZnT2-HA. GM means growth medium. Bars represent the fluorescence as percent of ZnT2-HA fluorescence. Error bars represent S.D. obtained from at least three independent experiments. Asterisks indicate that the values obtained are significantly higher than WT ZnT1 + ZnT2-HA *(t*-test with FDR, *α* = 0.05)

Flow cytometry-based quantification of zinc levels in cells transiently co-overexpressing WT or LoF mutant ZnT1 along with ZnT2-HA, showed that transient overexpression of exogenous WT-ZnT1 significantly decreased intracellular zinc levels, relative to the empty vector control (Fig. 5). Cells overexpressing mutant ZnT1 have increased intracellular zinc accumulation, relative to cells that overexpressed WT-ZnT1, as evidenced by the increased FluoZin3 fluorescence levels (Table S4). The intracellular zinc levels in cells transfected with the various ZnT1 mutants were similar to the levels of cells transfected with empty vector control, indicating that these mutants failed to extrude zinc from the cytoplasm. The Ruby fluorescence signal, did not differ between transfectants indicating comparable transfection efficiencies in the WT- and ZnT1 mutants, excluding V252G-ZnT1 which displayed lower fluorescence levels (Fig. S6, Table S5). The empty vector Ruby control had a lower mean Ruby fluorescence level due to its cytosolic localization, and therefore the fluorescence signal was decreased (Fig. S6). Collectively, the confocal microscopy imaging and the flow cytometry-based zinc transport assay, indicate that both overexpression of WT ZnT1 and LoF ZnT1 mutations lead to impaired intracellular zinc homeostasis.

### Increased MT-2 mRNA levels in ZnT1 mutant transfectants upon addition of exogenous zinc

**Fig. 6:**
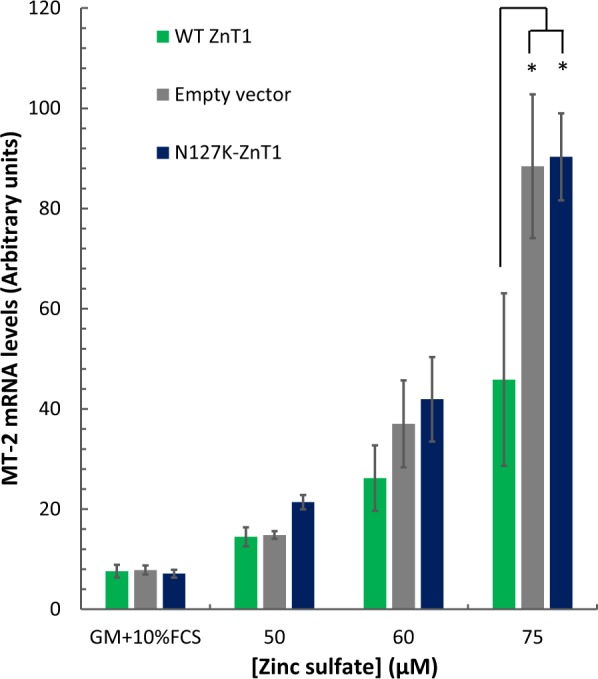
ZnT1 overexpression alleviates MT-2 response to elevated zinc. MT-2 mRNA levels in HEK-293 cells transfected with WT ZnT1, N127K-ZnT1 mutant, and empty vector control, upon treatment with RPMI-1640 growth medium (10% FCS), which contains ~2 µM zinc, or an additional 50 µM, 60 µM, or 75 µM zinc sulfate in the same medium. Results were obtained from at least 3 independent experiments. Asterisks indicate significantly higher MT-2 levels than WT ZnT1 in the same zinc concentration (*t* test with FDR, *α* = 0.05)

We further theorized that upon addition of exogenous zinc, which leads to increased intracellular zinc levels, cells transfected with LoF ZnT1 mutations but not with WT ZnT1, will upregulate alternative pathways to buffer the increased intracellular zinc levels. To examine alternative cellular protective pathways against increased intracellular zinc levels, we determined the mRNA levels of MT-2, a non-selective zinc metalloprotein, which was shown to sequester excess zinc^5^. We hypothesized that, upon exogenous zinc treatment, MT-2 mRNA levels will be upregulated in cells transfected with a representative mutant N127K-ZnT1, relative to cells transfected with WT ZnT1, due to the inability of the mutant ZnT1 to protect cells from zinc toxicity. Cells transfected with WT ZnT1, N127K-ZnT1, or empty vector control, were incubated with 50, 60, and 75 µM zinc sulfate and qRT-PCR was performed to determine relative MT-2 mRNA levels (Fig. 6). WT ZnT1 exerts a zinc protective function at the various zinc concentrations as indicated by the lower mRNA levels of MT-2 when compared to cells transfected with an empty vector or LoF mutant N127K-ZnT1 (Fig. 6). Upon addition of 75 µM zinc sulfate, empty vector and N127K-ZnT1 transfectants displayed an 11-fold and 13-fold increase in MT-2 mRNA levels, respectively, whereas cells transfected with WT-ZnT1 had only a sixfold increase in MT-2 mRNA levels (Fig. 6). At lower zinc concentrations, MT-2 mRNA levels did not vary significantly between the WT-ZnT1, N127K-ZnT1, and empty vector control, indicating that a threshold of extracellular zinc level is necessary to activate zinc protective pathways. These results suggest that cells expressing mutant ZnT1 proteins fail to extrude excess cytosolic zinc and hence other protective pathways against zinc toxicity are activated, such as increased metallothionein gene expression, in order to compensate for the loss of ZnT1 function.

## Discussion

The initial shift of a healthy cell towards malignant transformation occurs upon accumulation of cancer driver mutations in oncogenesis driver genes, which confer a selective growth advantage^34^. Driver mutations in proto-oncogenes like KRAS, are gain-of-function mutations, whereas tumor suppressor genes, such as TP53, harbor LoF mutations^34^. Due to genomic instability, cancer cells accumulate secondary passenger mutations, which are not causative of oncogenesis, cancer progression or metastasis^34^. Identification of potential novel cancer driver mutations, and their differentiation from passenger mutations, may enhance our understanding of the molecular mechanisms underlying initiation of oncogenesis, cancer progression and metastasis. In this respect, using mutation effect analyses, we herein showed an increased frequency of LoF ZnT1 mutations in cancer specimens. We further found that ZnT1 overexpression is common in various tumors and can predict patients’ survival. In order to obtain a better insight regarding the possible role of LoF ZnT1 mutations in cancer initiation and progression, and in order to render these LoF ZnT1 mutations potential cancer driver mutations, we explored the impact of these mutations on cellular zinc homeostasis in non-malignant human embryonic kidney HEK293 cells which harbor an intact zinc homeostasis. Using a novel functional zinc transport assay based on transient co-transfection of ZnT1 and ZnT2, we showed that overexpression of WT ZnT1 caused decreased intracellular zinc levels in HEK293 cells, whereas overexpression of LoF ZnT1 mutants failed to export zinc, resulting in zinc accumulation within intracellular vesicles. Loss of zinc transport in the ZnT1 mutants is likely due to the destabilization of the transmembrane region in the predicted ZnT1 structure. According to the predicted structure of ZnT1, mutagenesis of the residues selected for functional validation is detrimental for structural stability of the transmembrane region as well as for zinc transport function. Structural overlap of site A of zinc binding between ZnT1 and ZnT2 validates the accuracy of the ZnT1 structural prediction from a functional zinc transport standpoint.

**Fig. 7:**
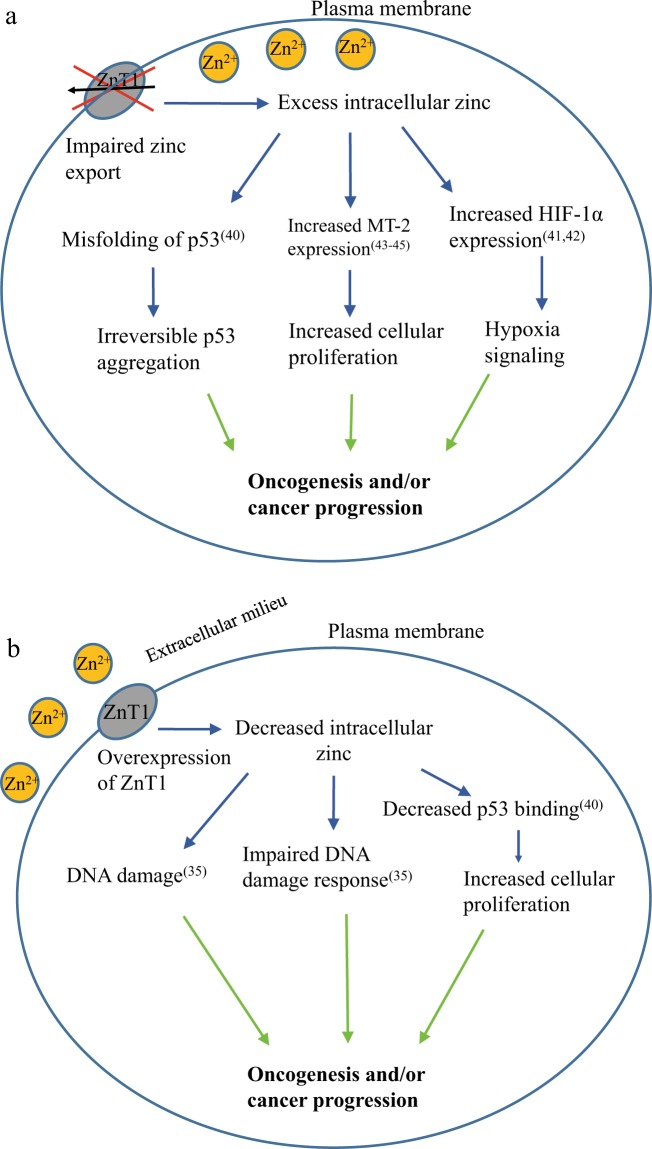
Published major molecular mechanisms that contribute to pro-oncogenic pathways by the virtue of impaired zinc homeostasis. **a** ZnT1 inactivating mutations increase intracellular zinc. **b** ZnT1 overexpression leads to decreased intracellular zinc

Comparative analysis of mRNA levels of ZnT1 in tumor specimens and healthy individuals revealed that there is a significant ZnT1 overexpression in specimens from various cancers including ESCA, thymoma, PAAD, READ, and STAD (Fig. 2). Importantly, we found that ZnT1 expression levels predicted decreased patient survival in the aforementioned cancers. Both low and high levels of intracellular zinc were previously shown to activate pro-oncogenic pathways and eventually lead to cancer development and cancer progression. For example, Ho and Ames showed that low intracellular zinc levels in glioma cells causes DNA damage, impaired DNA repair pathways, and a decrease in DNA target binding by p53, as well as by APE and NFkB, which are involved in DNA damage repair^35^. Therefore, we propose that ZnT1 overexpression activates pro-oncogenic pathways due to DNA damage, impaired DNA repair, and inhibition of p53 activity (Fig. 7a). In addition, zinc plays a key role in cell proliferation and differentiation, antioxidant defense, transcription factors and enzyme activity, apoptosis, and cell cycle regulation^3^. Impairment of these pathways due to zinc deficiency may lead to loss of critical cellular functions and promotion of pro-oncogenic processes^36^. A previous study showed that ZnO nanoparticles lead to increased p53 protein levels and induction of apoptosis in HepG2 cells^37^. Another study showed decreased VEGFR expression, decreased tubule formation, and increased apoptosis in endothelial colony-forming cells as a result of ZnO treatment^38^. Interestingly, a similar effect is observed with ZnCl_2_ treatment, indicating the therapeutic potential of zinc in ZnO nanoparticles^38^. Moreover, upregulation of ZnT1 was found in gastric cancer cells that acquired resistance to cisplatin^39^. Hence, ZnT1 overexpression may contribute to chemo-resistance of cancer cells, and serve as a potential new target in cancer therapeutics.

Apart from the major impact of ZnT1 overexpression in cancer, we showed here that the increase in LoF ZnT1 mutations in cancer specimens, resulting in the decrease in zinc efflux, leads to increased intracellular zinc levels (Fig. 5), as well as to elevated MT-2 mRNA levels upon addition of exogenous zinc (Fig. 6). We therefore propose that expression of these LoF ZnT1 mutations may induce several molecular mechanisms favorable for oncogenesis and cancer progression, as a result of increased intracellular zinc (Fig. 7b). In this respect, it was previously shown that the native conformation of p53 is zinc-dependent, and excess intracellular zinc causes p53 misfolding and aggregation^40^. We therefore propose that LoF mutations in ZnT1 lead to impaired zinc homeostasis resulting in p53 misfolding, thereby possibly contributing to oncogenesis. Increased intracellular zinc may also shift cells towards a state of oncogenesis via an additional layer of transcriptional regulation. For example, in MCF-7 cells, overexpression of HIF-1α, a hypoxia-induced transcriptional regulator, was observed upon exogenous zinc treatment^41^. Elevated levels of HIF-1α have been observed in tumor biopsies, and there was a strong correlation between HIF-1α levels, tumor angiogenesis, metastasis, and anticancer drug resistance^42^. Expression of ZnT1 mutations, which leads to impaired zinc homeostasis, may enhance tumor progression as a result of upregulation of key cellular pathways in hypoxia signaling.

We further showed that, when compared to cells transfected with the WT ZnT1, expression of a representative LoF ZnT1 mutation, N127K-ZnT1, and the addition of exogenous zinc, provoked high levels of MT-2 mRNA, which has been proposed as a cancer prognosis marker^43^. A clinical study showed elevated MT-2 levels in colorectal, breast, renal, and other tumor types^44^. Regardless of ZnT1 mutations, MT-2 has previously been suggested to modulate zinc-dependent transcription factors, such as Sp1 and p53 which may possibly be another pathway to induce promote cancer progression^45^.

We herein discussed the possible mechanisms that may support pro-oncogenic pathways by both decreased and increased intracellular zinc levels, caused by ZnT1 overexpression or by LoF mutations in ZnT1, respectively, and the subsequent alterations in ZnT1 activity. Previous studies have shown decreased plasma zinc levels in patients harboring bronchogenic carcinoma, lung cancer and leukemia^46,47^. However, systemic zinc deficiency was shown to inhibit tumor growth in rats^48^. Additionally, elevated zinc levels were reported within tumors, depending on the specific tumor type, with brain tumors displaying high zinc accumulation, as well pancreatic cancer samples that were found with high zinc concentrations^49,50^. In contrast, prostate cancer cells exhibit low levels of zinc when compared to cognate normal prostate tissues^51^. These findings suggest that both high and low zinc concentration states occur in tumors as well as in the tumor microenvironment, thereby supporting our hypothesis that dysregulation of zinc homeostasis, to higher or lower zinc levels, can possibly contribute to pro-oncogenic processes and cancer progression.

Differential expression of zinc transporters was previously reported in certain types of cancers. Prostate cells accumulate high levels of zinc for their normal function in citrate secretion into the prostatic fluid^51^. These high levels of zinc were suggested to have a protective role against prostate cancer. Decreased levels of ZIP1, which is responsible for zinc uptake into prostate cells, were found in both prostate tumors, and prostate cancer-derived cell lines^51^. Furthermore, increased ZnT1 mRNA levels were found in prostate cancer tissues, independent of tumor progression stage. In contrast to prostate cancer, pancreatic cancer specimens were found to overexpress ZIP4, when compared to the surrounding normal tissue^49^. Upregulation of ZIP4, a zinc influx transporter, leads to higher levels of zinc in these pancreatic cancer cells^49^. Moreover, ZIP10 expression was found to be higher in invasive and metastatic breast cancer cell lines, when compared to less invasive ones^52^. Zinc and ZIP10 depletion was shown to inhibit migration of metastatic cells lines suggesting a role of zinc in the metastatic phenotype of breast cancer cells^52^. The observed variation in zinc transporter expression in the different tumors is another layer of evidence for dysregulation of zinc levels in these malignant tissues, suggesting that zinc transporters may serve as a potential target for diagnosis, prognosis and/or cancer therapy. In this study we provide new evidence for abundant aberrant expression and function of ZnT1 in tumor specimens. This is specifically important as ZnT1 is the sole, functionally non-redundant zinc exporter, localized at the plasma membrane, and therefore has a central role in maintenance of intracellular zinc homeostasis. As discussed above, zinc homeostasis has a key role in a multitude of cellular functions, and dysregulation of zinc homeostasis leads to cancer and/or cancer progression. Thus, as a primary regulator of zinc homeostasis, ZnT1 may prove a cancer driver gene and a possible druggable target.

## Supplementary information

Supplementary figure legends

Figure S1: Pairwise Alignment between ZnT1 and the YiiP template (PDB 3j1z), computed by RaptorX algorithm and manually improved

Figure S2: Mutation annotation analysis workflow. For ZnTs and ZIPs, exome sequences in the gnomAD database, as well as tumor sample sequences from the COSMIC database, were analyzed via the variant effect predictor, in order to annotate the predicted percentage of deleteriousness of missense mutations

Figure S3: Inactivating mutations in ZIP zinc transporters are more abundant in cancer as compared to healthy controls. Odds ratio (black dot) of (a) LoF mutations and (b) predicted deleterious missense mutations, identified in ZIP1-ZIP14 in tumor samples (COSMIC) versus healthy controls (gnomAD). Error bars represent 0.95 confidence interval

Figure S4: GEPIA expression boxplot representation of ZnT1 expression in KICH cancer type and control. Asterisk indicates p-value < 0.005 (ANOVA). KICH-Kidney Chromophobe

Figure S5: Predictive topology of ZnT1 was done using Protter software, with transmembrane regions predefined by TOPCONS predictions, a conglomerate of TM region predictive tools yielding higher accuracy. The amino acids circled in green represent the residues in the conserved zinc-binding domain. The residues circled in purple represent charged predicted TM residues, while the residues circled in red are the ones we chose for functional validation

Figure S6: Ruby fluorescence intensity of HEK-293 cells co-transfected with mutant ZnT1 as well as ZnT2 (relative to ZnT1-WT and ZnT2-HA), indicates that transfection efficiency among WT ZnT1 and mutant ZnT1 is similar. Bars represent the fluorescence as percent of ZnT1WT+ZnT2-HA fluorescence. Error bars represent S.D. of at least 3 independent experiments. Asterisks indicate that the values obtained are significantly higher than WT-ZnT1+ZnT2-HA (t test with FDR, α=0.05)

Table S1: Primers for site-directed mutagenesis of ZnT1 residues from Table 1

Table S2: Number of conspicuous LoF mutations and missense mutations in ZnT1-ZnT10 in COSMIC and gnomAD, their odds ratio, and p-values. Asterisks indicate statistically significant increase in LoF or predicted deleterious missense mutations in tumor specimens, compared to healthy controls(t test with FDR, α=0.05)

Table S3: Number of conspicuous LoF mutations and missense mutations in ZIP1-ZIP14 in COSMIC and gnomAD, their odds ratio, and p-values. Asterisks indicate statistically significant increase in LoF or predicted deleterious missense mutations in tumor specimens, compared to healthy controls (t test with FDR, α=0.05)

Table S4: FluoZin3 raw data for Figure 5

Table S5: Ruby fluorescence levels for Figure S3
