# Supplementary material for: Alterations in ZnT1 expression and function lead to impaired intracellular zinc homeostasis in cancer
Source: Cell Death Discov. 2019 Nov 12;5:144. doi: 10.1038/s41420-019-0224-0 (PMC6851190; doi:10.1038/s41420-019-0224-0)
Supplement: Supplementary file 1 — Supplementary figure legends [file 41420_2019_224_MOESM1_ESM.docx]

**Supplementary Figure legends**

**Figure S1:** Pairwise Alignment between ZnT1 and the YiiP template (PDB 3j1z), computed by RaptorX algorithm and manually improved.

**Figure S2:** Mutation annotation analysis workflow. For ZnTs and ZIPs, exome sequences in the gnomAD database, as well as tumor sample sequences from the COSMIC database, were analyzed via the variant effect predictor, in order to annotate the predicted percentage of deleteriousness of missense mutations.

**Figure S3:**  **Inactivating mutations in ZIP zinc transporters are more abundant in cancer as compared to healthy controls.** Odds ratio (black dot) of **(a)** LoF mutations and **(b)** predicted deleterious missense mutations, identified in ZIP1-ZIP14 in tumor samples (COSMIC) versus healthy controls (gnomAD). Error bars represent 0.95 confidence interval.

**Figure S4:** GEPIA expression boxplot representation of ZnT1 expression in KICH cancer type and control. Asterisk indicates p-value < 0.005 (ANOVA). KICH-Kidney Chromophobe

**Figure S5**: Predictive topology of ZnT1 was done using Protter software, with transmembrane regions predefined by TOPCONS predictions, a conglomerate of TM region predictive tools yielding higher accuracy. The amino acids circled in green represent the residues in the conserved zinc-binding domain. The residues circled in purple represent charged predicted TM residues, while the residues circled in red are the ones we chose for functional validation.

**Figure S6:** Ruby fluorescence intensity of HEK-293 cells co-transfected with mutant ZnT1 as well as ZnT2 (relative to ZnT1-WT and ZnT2-HA), indicates that transfection efficiency among WT ZnT1 and mutant ZnT1 is similar. Bars represent the fluorescence as percent of ZnT1WT+ZnT2-HA fluorescence. Error bars represent S.D. of at least 3 independent experiments. Asterisks indicate that the values obtained are significantly higher than WT-ZnT1+ZnT2-HA (t test with FDR, α=0.05).

**Supplementary Tables**

**Table S1:** Primers for site-directed mutagenesis of ZnT1 residues from Table 1.

**Table S2:** Number of conspicuous LoF mutations and missense mutations in ZnT1-ZnT10 in COSMIC and gnomAD, their odds ratio, and p-values. Asterisks indicate statistically significant increase in LoF or predicted deleterious missense mutations in tumor specimens, compared to healthy controls (t test with FDR, α=0.05).

**Table S3:** Number of conspicuous LoF mutations and missense mutations in ZIP1-ZIP14 in COSMIC and gnomAD, their odds ratio, and p-values. Asterisks indicate statistically significant increase in LoF or predicted deleterious missense mutations in tumor specimens, compared to healthy controls (t test with FDR, α=0.05).

**Table S4:** FluoZin3 raw data for Figure 5.

**Table S5:** Ruby fluorescence levels for Figure S3
