# Supplementary figures and images for: Alterations in ZnT1 expression and function lead to impaired intracellular zinc homeostasis in cancer
Source: Cell Death Discov. 2019 Nov 12;5:144. doi: 10.1038/s41420-019-0224-0 (PMC6851190; doi:10.1038/s41420-019-0224-0)

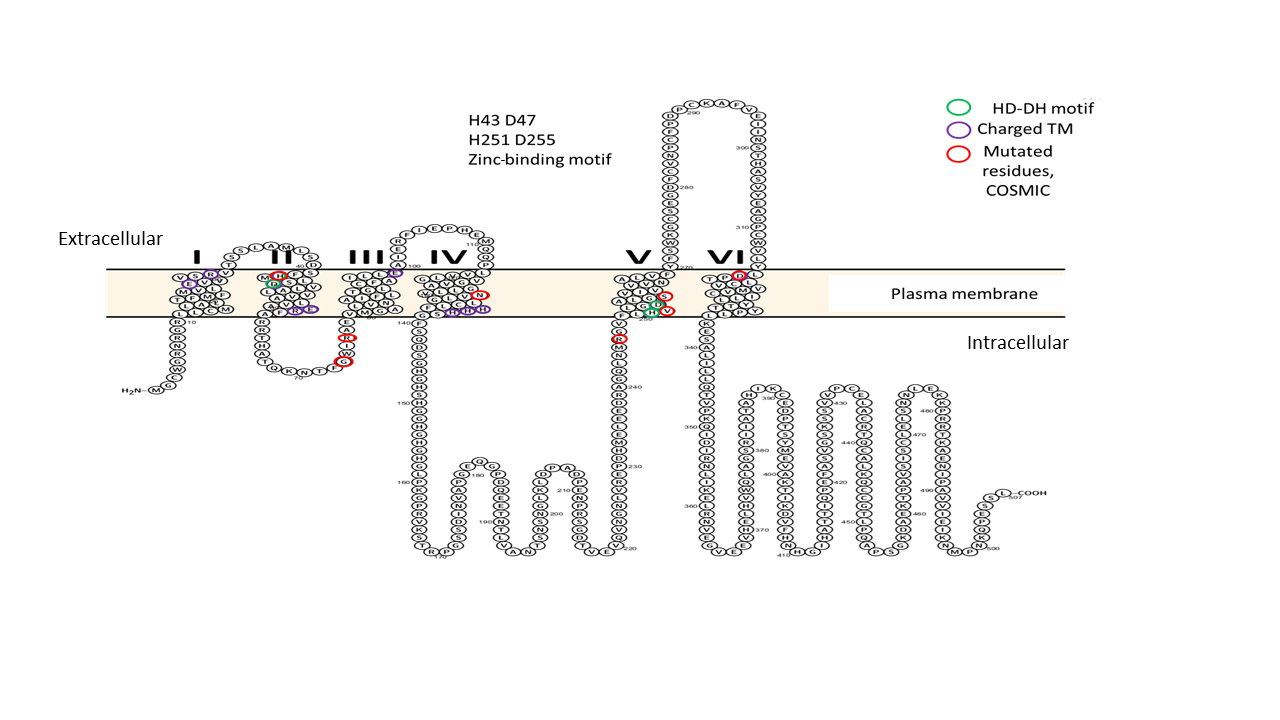

Supplement: Supplementary file 6 — Figure S5: Predictive topology of ZnT1 was done using Protter software, with transmembrane regions predefined by TOPCONS predictions, a conglomerate of TM region predictive tools yielding higher accuracy. The amino acids circled in green represent the residues in the conserved zinc-binding domain. The residues circled in purple represent charged predicted TM residues, while the residues circled in red are the ones we chose for functional validation [file 41420_2019_224_MOESM6_ESM.tif]
