# Supplementary material for: Alterations in ZnT1 expression and function lead to impaired intracellular zinc homeostasis in cancer
Source: Cell Death Discov. 2019 Nov 12;5:144. doi: 10.1038/s41420-019-0224-0 (PMC6851190; doi:10.1038/s41420-019-0224-0)
Supplement: Supplementary file 8 — Table S1: Primers for site-directed mutagenesis of ZnT1 residues from Table 1 [file 41420_2019_224_MOESM8_ESM.docx]

| **residue** | **Forward** | **Reverse** |
| --- | --- | --- |
| **D47G** | CACATGCTGTCGGGCGTGCTGGCACTGGTGGTGGCG | CGCCACCACCAGTGCCAGCACGCCCGACAGCATGTG |
| **G73D** | CAGAAGAACACGTTCGACTGGATCCGAGCCG | CGGCTCGGATCCAGTCGAACGTGTTCTTCTG |
| **R76L** | GTTCGGCTGGATCCTAGCGGAGGTAATGGGGGC | GCCCCCATTACCTCCGCTAGGATCCAGCCGAAC |
| **N127K** | GCTGCTGGTCAAGGTGCTGGGGCTCTG | CAGAGCCCCAGCACCTTGACCAGCAGC |
| **R246C** | GCTGGACAACTTAACATGTGTGGAGTTTTTCTGC | GCAGAAAAACTCCACACATGTTAAGTTGTCCAGC |
| **V252G** | GTGGAGTTTTTCTGCATGGCCTTGGAGATGCCTTG | CAAGGCATCTCCAAGGCCATGCAGAAAAACTCCAC |
| **S259L** | GTCCTTGGAGATGCCTTGGGTTTAGTGATTGTAGTAGTAAATGCC | GGCATTTACTACTACAATCACTAAACCCAAGGCATCTCCAAGGAC |
| **D317Y** | CCTTGCTGGGTGCTATATTTATATCCAACTCTTTGTGTTGTAATG | CATTACAACACAAAGAGTTGGATATAAATATAGCACCCAGCAAGG |

Table S1
